# Isolation and Determination of Fomentariol: Novel Potential Antidiabetic Drug from Fungal Material

**DOI:** 10.1155/2018/2434691

**Published:** 2018-02-20

**Authors:** Nevena Maljurić, Jelena Golubović, Matjaž Ravnikar, Dušan Žigon, Borut Štrukelj, Biljana Otašević

**Affiliations:** ^1^Department of Drug Analysis, Faculty of Pharmacy, University of Belgrade, Vojvode Stepe 450, Belgrade 11221, Serbia; ^2^Chair for Pharmaceutical Biology, Faculty of Pharmacy, University of Ljubljana, Aškerčeva cesta 7, Ljubljana 1000, Slovenia; ^3^Jožef Stefan Institute, Jamova 39, Ljubljana 1000, Slovenia

## Abstract

Diabetes mellitus is one of the leading world's public health problems. Therefore, it is of a huge interest to develop new antidiabetic drugs. Apart from traditional therapy of diabetes, nowadays, importance is given to natural substances with antidiabetic potential. *Fomes fomentarius* is a mushroom widely used for different purposes, due to its range of already confirmed activities. Fomentariol is a constituent of *Fomes fomentarius*, responsible for its antidiabetic potential. In that respect, it is important to develop a method for isolation and quantification of fomentariol from fungal material, which will be simple and efficient. Multistep, complex extraction applied in the previously reported studies was avoided with ethanol, providing rapid single-step extraction. The presence of fomentariol in ethanolic extract was confirmed by high-resolution mass spectrometry. Semipreparative HPLC method was developed and applied for isolation from ethanol extract and purification of the active compound fomentariol. It was a gradient reversed-phase method with a mobile phase consisting of acetonitrile and 0.1% formic acid in water and total run time of 15 minutes. The amount of 6.5 mg of high-purity fomentariol was determined by quantitative NMR with toluene as internal standard. The isolated and determined amount of substance can be further used for the quantitative estimation of activity of fomentariol.

## 1. Introduction

*Fomes fomentarius* is a mushroom of the family Polyporaceae, native to the north of the temperate zone of the northern hemisphere. Although this mushroom is firstly described in the 5th century BC by Hippocrates and has been traditionally used worldwide for different purposes, not much is published about its medicinal usage. Recent studies demonstrated that extracts of *F. fomentarius* exerted antidiabetic, antioxidant, anti-inflammatory, antinociceptive, antibacterial, and cytotoxic activities, either by unknown mechanisms [1, 2] or by virtue of the active principles other than fomentariol [3, 4].

**Figure 1:**
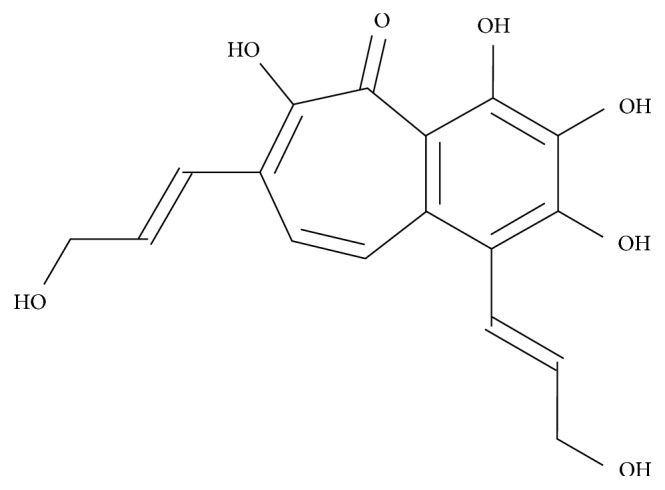
Structure of fomentariol.

Fomentariol (Figure 1) is a constituent of *F. fomentarius*. Although it was recognized long ago [5], its activity is insufficiently investigated. Seo et al. [6] isolated fomentariol and demonstrated its antioxidant activity.

On the other hand, our experiments with *α*-glucosidase and dipeptidyl peptidase-4 showed antidiabetic potential of fomentariol. Diabetes mellitus is a major public health problem that is approaching epidemic proportions globally. Development of new antidiabetic drugs is of huge interest to mankind. Natural substances with antidiabetic potential can be a very valuable alternative or supplement to the conventional therapy of diabetes.

The purpose of this study was to develop a simple and efficient method for the isolation and determination of fomentariol from the fungal material, in order to quantify its antidiabetic activity, that is, to determine its EC50. Reference standard substance is not commercially available, and the synthesis would be quite challenging [7]. Complementary analytical methods, such as mass spectrometry (MS), high-performance liquid chromatography (HPLC), and nuclear magnetic resonance spectroscopy (NMR), can be applied in order to confirm identity, obtain high-purity substance, and determine the quantity of the isolated substance, respectively.

Seo et al. [6] only investigated the mechanism of its antioxidant activity, without quantitative examination of this activity. Furthermore, extraction and isolation of fomentariol in the previous studies published by Seo et al. [6] consisted of multistep extraction employing several solvents, followed by several different chromatographic procedures. First isolation of fomentariol from the natural source also included multisolvent extraction [5]. Therefore, the important goal of this paper was also to propose a new procedure in order to simplify the extraction and isolation as much as possible, retaining the high efficiency.

The developed method must be robust and easily applied by analysts, allowing isolation and quantification of the compound in a reasonable time period.

## 2. Materials and Methods

### 2.1. Chemicals and Reagents

Ethanol (99.5%) used for the extraction of fomentariol was obtained from Sigma-Aldrich Chemie GmbH (Taufkirchen, Germany), while dichloromethane (99.8%) was obtained from Honeywell Riedel-de Haën (Seelze, Germany). Acetonitrile (99.8%) and formic acid (98%) were also purchased from Sigma-Aldrich Chemie GmbH, as well as methanol-d4 (99.9%). Toluene (99.5%) obtained from POCH (Gliwice, Poland) was used for quantitative NMR determination. Purified water was obtained from a Simplicity 185 purification system (Millipore, Billerica, MA, USA). Before use, the sample was filtered through 0.22 *µ*m nylon membranes (Agilent Technologies, Santa Clara, USA). All reagents used were of analytical grade except water and acetonitrile, which were of HPLC grade.

### 2.2. Identification and Isolation of Fomentariol

#### 2.2.1. Sample Preparation

Fungal material was characterized and kindly provided by Professor Franc Pohleven from the Department of Wood Science and Technology at the Biotechnical Faculty, University of Ljubljana. The fungal material was chopped and added to 50 mL of ethanol. Ethanolic extract was incubated for 24 hours at room temperature. After 24 hours, the extract was paper filtered. In order to evaporate the solvent, the extract was left overnight at room temperature. Dry extract was reconstituted in the solvent which consisted of acetonitrile and water (50  :  50, v/v) and was filtered through a 0.22 *µ*m nylon filter.

#### 2.2.2. Identification by Means of High-Resolution Mass Spectrometry Analysis

Fomentariol was identified by mass measurements run on a hybrid quadrupole time-of-flight mass spectrometer Q-TOF Premier provided with an orthogonal Z-spray ESI interface (Waters Micromass, Manchester, UK). Mass spectrometer was coupled to Waters Acquity ultra-high-performance liquid chromatography (UPLC) (Waters, Milford, USA) system based on a binary pump. The separation was achieved on Luna^®^ Omega C18 HPLC column (1.6 *µ*m, 100 × 2.1 mm i.d., Phenomenex Inc., USA), with temperature set to 40°C. Compressed nitrogen (99.999%, Messer Slovenia) was used as both the drying and the nebulising gas. The nebulizer gas flow rate was set to approximately 20 L/h and the desolvation gas flow rate to 600 L/h. A cone voltage of 20 V and a capillary voltage of 3.0 kV were used in positive ion mode, while 2.5 kV was used in negative ionization mode. The desolvation temperature was set to 300°C and the source temperature to 100°C. Elemental composition was determined with the mass resolution of approximately 9000 full width of the peak at half its maximum (FWHM) height. MS spectra were acquired in centroid mode over an m/z range of 50–1000 in scan time 0.2 s and interscan time 0.025 s. For MS/MS experiments, argon (99.995%, Messer Slovenia) was used as collision gas at a pressure of approximately 2 × 10^−5^ mbar in the collision cell. Collision energies of 15 V were applied to generate product ion spectra. MS/MS spectra were acquired in centroid mode as well, over the same m/z range and scan time. The detector potential was set to 2100 V. The mobile phase consisted of acetonitrile (A) and 0.1% formic acid in water (B). The gradient started with 95% B, which was decreased to 5% in 6 min and returned to initial ratio in 0.05 min, followed by re-equilibration, giving a total run time of 7 min. The flow rate was 0.3 mL·min^−1^. The operating software MassLynx v 4.1 (Waters Micromass, Manchester, UK) was used for data analysis.

#### 2.2.3. Isolation of Fomentariol

The isolation of fomentariol was performed on its ethanol extract on *Dionex Ultimate 3000* HPLC system, equipped with a PDA detector. Chromatographic separation was achieved on Hypersil Gold semipreparative HPLC column (Thermo Fisher Scientific Inc., 5 *µ*m, 150 × 10 mm). Injection volume was 100 *μ*L. The mobile phase consisted of acetonitrile (A) and 0.1% formic acid in water (B). The HPLC method was the one applied for the identification, only transferred from UPLC to semipreparative conditions. The gradient started with 95% B, which was decreased to 5% in 13 min, and returned to initial ratio in 0.05 min, followed by re-equilibration, giving a total run time of 15 minutes. The flow rate was 2 mL·min^−1^. The detection was performed at 326 nm, and UV-Vis spectra of the major peaks were recorded. Fractions were collected according to the retention of fomentariol, at the time frame 9.3–10 min.

### 2.3. Quantitative NMR Determination

The solvent in the collected fractions of fomentariol was evaporated to dry using Rotavapor R-114 (Büchi, Flawil, Switzerland), and the dry sample was dissolved in methanol-d4 (99.9%). The NMR spectra were recorded on a Bruker Ascend 400 (400 MHz) spectrometer (Billerica, USA). Chemical shifts are given in parts per million (*δ*) downfield from tetramethylsilane as the standard used for system calibration. The quantity of the compound was calculated by the relative ratio of the integral values of the target peaks of fomentariol to the ones of toluene, internal standard of known amount.

## 3. Results and Discussion

### 3.1. Extraction Optimization

Firstly, there are certain prerequisites in order to preserve the activity of the mushroom. The mushroom needs to be fresh, although it could be used for some period of time if it is kept frozen. When analyzing the potential activity of the mushroom components, the first step is to choose the right extraction solvent, suitable for the active compound. Our goal was to find a single solvent suitable for the extraction and avoid multistep, complex extraction applied in the previous studies [2, 5, 6]. The first try was with dichloromethane, which resulted in a poor extraction. Ethanol provided much more efficient extraction. The extraction efficiency was estimated by means of UPLC-MS method, which will be described in the next chapter. We noticed that the color of the extract can serve as a simple screening test, since the ethanol extract was orange to red colored, indicating the presence of fomentariol [3]. Furthermore, ethanol can be a convenient solvent for the future formulation of the drug product.

### 3.2. Identification of Fomentariol by High-Resolution Mass Spectrometry

**Figure 2:**
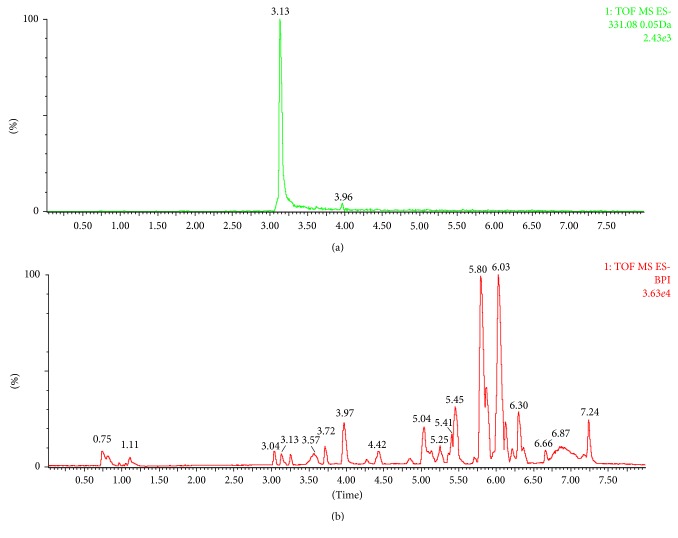
Extracted ion chromatogram of [M-H]^−^ of fomentariol at m/z 331 (a) and total ion chromatogram (b) of the *F. fomentarius* ethanolic extract.

**Figure 3:**
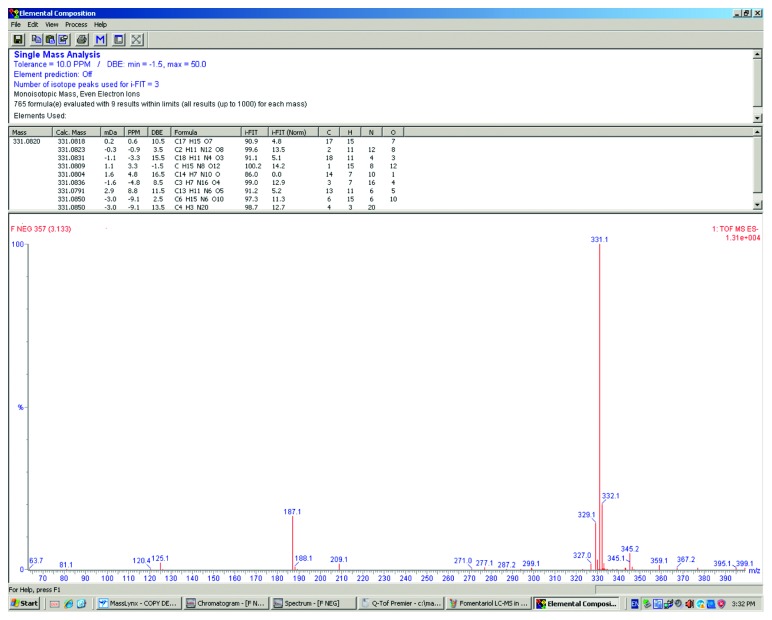
Electrospray ionization mass spectrum of fomentariol in negative ionization mode and results of mass measurement for elemental composition of [M-H]^−^ at m/z 331.

**Figure 4:**
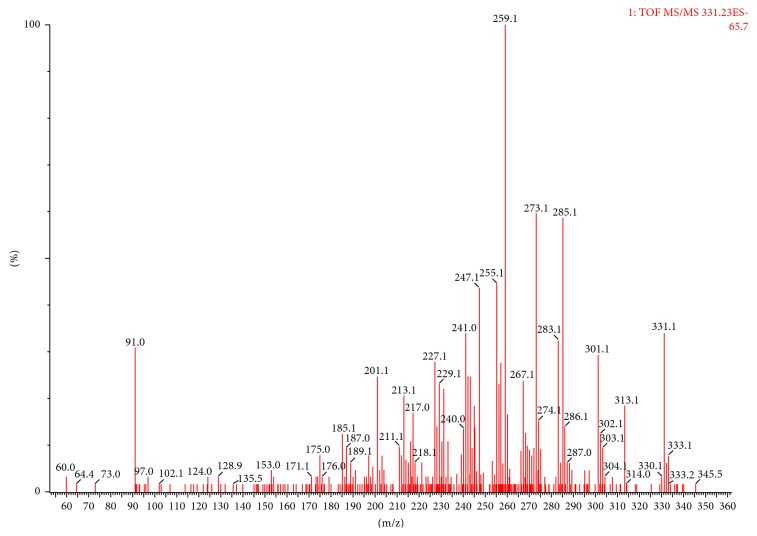
Product ion spectrum under the collision energy of 15 V.

The presence of fomentariol in ethanol extract was confirmed by high-resolution mass spectrometry (HRMS), comparing the obtained mass to charge ratio (m/z) with the theoretical one. When running UPLC-MS analysis, fomentariol peak was detected at 3.13 min retention time, as shown in Figure 2. Fomentariol was detected in both ionization modes, but higher signal intensity was obtained in the negative mode. Exact mass of the [M-H]^−^ ion under the peak of interest was m/z 331.0820. Elemental composition analysis proposed molecular formula of fomentariol as the first hit (Figure 3). This is also in accordance with the theoretical mass provided by ChemSpider®, which is 332.0896 Da. To our knowledge, no MS/MS spectra of fomentariol can be found in literature. We provided hereby the product ion spectrum under the collision energy of 15 V (Figure 4). All of these information combined served to confirm that the peak of interest corresponds to fomentariol.

### 3.3. Isolation of Fomentariol Using Semipreparative HPLC

**Figure 5:**
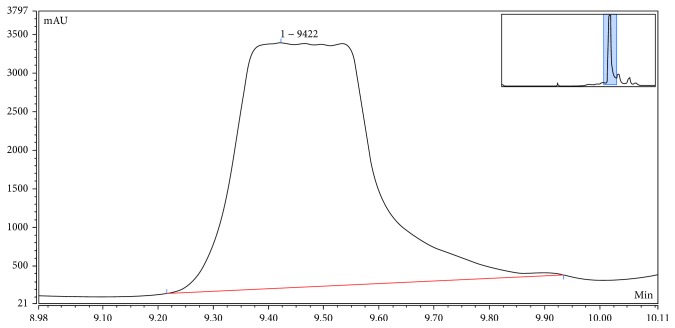
The zoomed peak of fomentariol under the semipreparative HPLC conditions.

Developing a simple and efficient method for isolation of fomentariol is crucial for its quantitative determination in order to access its activity. Fomentariol is a moderately polar substance, so a reverse-phase column was selected as the stationary phase, while gradient composition of acetonitrile and 0.1% formic acid in water was used as the mobile phase. UV-Vis spectra of the major peaks in chromatograms were recorded using PDA and used as a primary identification tool. The ethanol extract of the fungi was used to isolate fomentariol on preparative scale, using HPLC, as described. Semipreparative HPLC method was developed and applied for analyzing the ethanol extract of the fungi for the purpose of isolation and purification of the active compound fomentariol.  Figure 5 represents the zoomed peak corresponding to fomentariol. The peak had a flat-top shape, indicating the desired saturation of the column. Fractions were collected according to the retention of fomentariol, at the time frame 9.3–10.0 min. The collected fractions were further evaporated and used for the quantitative determination of fomentariol. Color intensity of the collected fractions was an additional confirmation of the accurate fraction collection.

### 3.4. Quantitative NMR Determination

In NMR analysis, the ratio of the number of atomic nuclei in a compound corresponds to the ratio of the areas of the peaks in the spectrum. Therefore, the unknown amount of the compound of interest can be determined by performing a quantitative analysis. When the amount is determined by ^1^H NMR, the sample is mixed with an internal standard having a known purity and dissolved in a deuterated solvent. The relationship between the areas of the spectral peaks originated from the sample and the standard, the number of protons, the weighed masses, and the molecular weights of the sample and the standard are used to calculate the quantitative value of the purity of the sample. In quantitative analysis using ^1^H NMR (quantitative NMR), the areas of the peaks of hydrogen atoms observed in the spectrum can be quantitatively compared. Therefore, it is possible and rather easy to determine the purity and quantity of many compounds containing a hydrogen atom with one standard. The only condition is that the signals of the sample and the standard do not overlap with each other [8–14].

**Figure 6:**
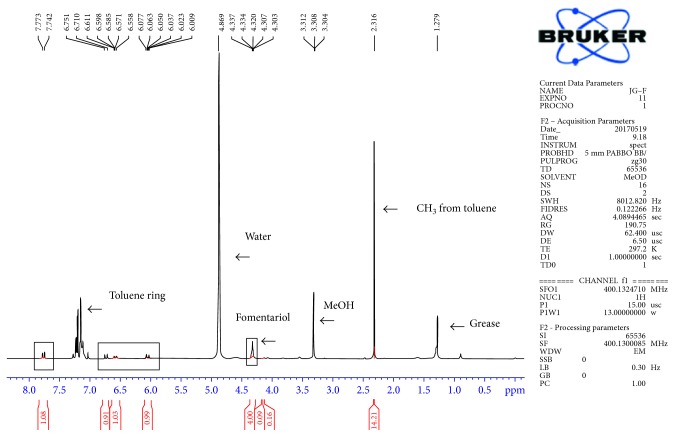
^1^H NMR spectrum of fomentariol and toluene as internal standard.

Since the target compound fomentariol is polar, methanol-d4 was used as the solvent for NMR analysis, to ensure that the compound will be completely dissolved. Quantification of fomentariol by ^1^H NMR is possible by means of the integral of a well-separated specific proton signal of the compound. NMR spectrum including the interpretation is presented in Figure 6. The integral value of the signal of fomentariol at 4.35 ppm was compared to the integral value of the internal standard signal. A suitable internal standard should preferably be a stable compound with a signal in a noncrowded region of the ^1^H NMR spectrum. For this purpose, toluene with a signal at 2.316 ppm has been chosen. In the case of ^1^H NMR quantitative analysis, there is no need for the construction of calibration curves in order to quantify the compounds, because integration of the peaks is always proportional to the amount of the compound. The amount of fomentariol in the sample was calculated knowing the amount of toluene. Three protons of 8.3 mg (0.0935 mmol) of toluene gave the integral value of 14.21. The amount of toluene in the mixture was 4.7 times higher than amount of fomentariol (14.21/3). The calculated amount of fomentariol in the sample was 0.0198 mmol, that is, 6.5 mg. Signals in the spectra which corresponded to the compounds other than the known ones indicated high purity of the sample, around 90%.

**Figure 7:**
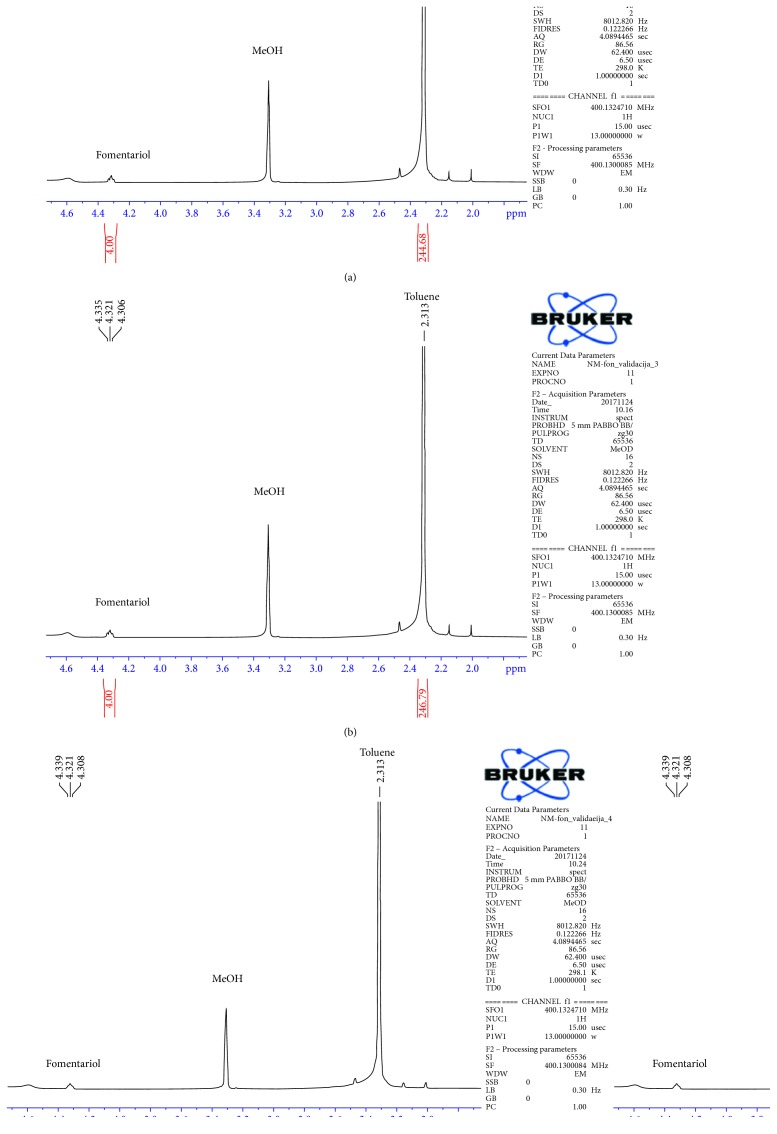
^1^H NMR spectra of fomentariol and toluene as internal standard recorded subsequently for the estimation of method reproducibility.

The reproducibility of the measurements was established through three replicate analyses of the sample of unknown amount of isolated fomentariol [11, 12]. The integration region for the signal of fomentariol was 4.28–4.36 ppm, while for the signal of toluene, used as internal standard, it was 2.29–2.35 ppm (Figure 7). The calculated amount of fomentariol over three measurements was 1.538 mg, 1.525 mg, and 1.525 mg, subsequently. Relative standard deviation (RSD) was calculated as a measure of precision. The obtained standard deviation (SD) was 0.006128, while calculated RSD was 4%. The obtained data show a good reproducibility of the measurement carried out by ^1^H NMR technique.

## 4. Conclusion

In the present paper, several analytical techniques were successfully combined in order to isolate and quantify fomentariol, as a potential antidiabetic natural drug from the mushroom *Fomes fomentarius*. The single-solvent extraction with ethanol appeared to have high efficiency. The substance of interest was confirmed by UPLC-HRMS. The UPLC method was transferred to semipreparative HPLC and applied for the collection of fractions corresponding to fomentariol. The amount of fomentariol in the collected fractions was determined by means of NMR spectroscopy method, with the required reproducibility. In that way, we obtained high-purity standardized fomentariol, which can be further used for the quantitative estimation of its activity.
